# Resting-state BOLD signal variability is associated with individual differences in metacontrol

**DOI:** 10.1038/s41598-022-21703-5

**Published:** 2022-11-01

**Authors:** Chenyan Zhang, Christian Beste, Luisa Prochazkova, Kangcheng Wang, Sebastian P. H. Speer, Ale Smidts, Maarten A. S. Boksem, Bernhard Hommel

**Affiliations:** 1 Cognitive Psychology Unit, Leiden Institute for Brain and Cognition, Institute of Psychology, Leiden University, Leiden, The Netherlands; 2 Department of Child and Adolescent Psychiatry, Faculty of Medicine, TU Dresden, Dresden, Germany; 3 University Neuropsychology Center, Faculty of Medicine, TU Dresden, Dresden, Germany; 4 School of Psychology, Shandong Normal University, Jinan, China; 5 Social Brain Lab, Netherlands Institute for Neuroscience, Amsterdam, The Netherlands; 6 Princeton Neuroscience Institute, Princeton University, Princeton, NJ, USA; 7 Rotterdam School of Management, Erasmus University Rotterdam, Rotterdam, The Netherlands

**Keywords:** Human behaviour, Neuroscience, Psychology

## Abstract

Numerous studies demonstrate that moment-to-moment neural variability is behaviorally relevant and beneficial for tasks and behaviors requiring cognitive flexibility. However, it remains unclear whether the positive effect of neural variability also holds for cognitive persistence. Moreover, different brain variability measures have been used in previous studies, yet comparisons between them are lacking. In the current study, we examined the association between resting-state BOLD signal variability and two metacontrol policies (i.e., persistence vs. flexibility). Brain variability was estimated from resting-state fMRI (rsfMRI) data using two different approaches (i.e., Standard Deviation (SD), and Mean Square Successive Difference (MSSD)) and metacontrol biases were assessed by three metacontrol-sensitive tasks. Results showed that brain variability measured by SD and MSSD was highly positively related. Critically, higher variability measured by MSSD in the attention network, parietal and frontal network, frontal and ACC network, parietal and motor network, and higher variability measured by SD in the parietal and motor network, parietal and frontal network were associated with reduced persistence (or greater flexibility) of metacontrol (i.e., larger Stroop effect or worse RAT performance). These results show that the beneficial effect of brain signal variability on cognitive control depends on the metacontrol states involved. Our study highlights the importance of temporal variability of rsfMRI activity in understanding the neural underpinnings of cognitive control.

## Introduction

Neural activity is highly variable from moment to moment at every level of neural organization. Traditionally, variability of this kind is considered to be “noise” that tends to mask, overshadow, or even distort the neural signals that are assumed to represent the relevant neural processing. Accordingly, functional magnetic resonance imaging (fMRI) research typically focuses on mean activity within a voxel or brain region, but considers variance in blood oxygen level-dependent (BOLD) signal as to-be-neglected “noise”^1^. The same logic applies to other neuroscientific and behavioral measurements indicative of human cognitive functioning^2^.

However, accumulating evidence suggests that intra-individual variability might be functional and beneficial for cognitive performance^3–8^, so that a better understanding of its functional role might strongly improve the diagnosis and treatment of mental disorders such as ADHD^9–12^. For example, higher BOLD signal variability is associated with younger age, higher accuracy, faster and more stable responses across a number of cognitive tasks spanning perception, attention, working memory, response inhibition and task switching^4,6,7,13–16^. BOLD signal variability might reflect intrinsic properties of network organization^8^, cardiovascular and cerebrovascular factors^17^, and/or general non-cognitive factors^18^. Notably, previous work suggests that more pronounced brain variability might allow the brain to explore among different functional network configurations, which in turn supports cognitive flexibility—the ability to explore variable opportunities and flexibly adapt to changing circumstances^5,16,19^.

The present study was motivated by the idea that individual differences in cortical variability might be systematically related to individual cognitive-control styles, to what Hommel (2015) has called “metacontrol”^20^. This term refers to the control of cognitive functioning to deal with a fundamental dilemma of human cognition^21–23^: the fact that we sometimes need to be “cognitively conservative” by sticking with our present mindset and our present goal, but to be flexible and more open to alternative goals on other occasions. Hence, people need both cognitive persistence and cognitive flexibility: while cognitive flexibility helps them to switch between alternative opportunities, intentional agents also need cognitive persistence to avoid distractions and to stick with the current goal as long as pursuing it is worthwhile^20,24–26^.

As Hommel’s Metacontrol State Model (MSM) suggests, cognitive control emerges from the interplay of two counteracting forces or systems, one promoting cognitive persistence and the other promoting cognitive flexibility^20^. A metacontrol bias towards persistence is characterized by a strong top-down influence from the current goal and restricting processing to task-relevant information. In contrast, a metacontrol bias towards flexibility is characterized by a stronger bottom-up influence and openness to alternative goals and opportunities^20^. Truly adaptive control requires humans to find a balance between persistence and flexibility, an ability called metacontrol. Interestingly, there are systematic individual differences with respect to the metacontrol default: while some people tend to have a persistence bias, so that they perform better than others on tasks that require persistence but less well than others on tasks that require flexibility, other individuals tend to have a flexibility bias, resulting in the opposite performance profile^26^. The basic idea driving the present study was that such individual biases in metacontrol might be related to individual differences in brain variability, that is, in the individual level of the BOLD signal variability of people’s brains.

We assessed our key hypothesis by testing whether an indicator of the individual degree of brain variability, our noise measure, is statistically correlated to behavior in tasks that have been shown to be diagnostic for individual biases towards metacontrol persistence or flexibility. “Noise” is defined as variability that results from random or unpredictable fluctuations and disturbances^27^. We used resting-state fMRI (rsfMRI) measures as indicators of the individual variability level. RsfMRI is a spontaneous low frequency (< 0.1 Hz) BOLD signal within the brain in the absence of external stimulation. Noise (at an optimal level) in rsfMRI is thought to drive the network dynamics^28,29^ and enables the exploration of the brain among various functional configurations representing its dynamic repertoire^19^. It thus seems possible that cortical noise is systematically related to metacontrol.

Various temporal variability estimation approaches for rsfMRI data have been introduced and used in previous studies^4,7,30,31^. The simplest and most prominent measure of variability is the standard deviation (SD), which reflects the distributional width of a BOLD signal time series. The SD of a BOLD signal is related to age and cognitive performance in both younger and older adults^4,7^. However, SD overestimates the true dispersion when the (mean) signal varies because the calculation of SD is based on the difference between single data points and the overall mean^32^. To circumvent this problem, some researchers have suggested an alternative measure—the mean squared successive difference (MSSD)^30,33,34^. The MSSD captures the BOLD signal difference between successive time points and thus can adapt to changing expected (mean) signals. Although the advantages and disadvantages of different measures have been discussed in the literature^7,30,33^, it is unknown whether different parameters that can be estimated on the basis of rsfMRI data reveal differences in their predictability to cognitive control. Given that we had no a-priori reason to favor one measure over another, we considered both of them, assuming that a systematic comparison would lay the grounds for choosing proper measurement approaches in future studies. Therefore, the present study employed two different brain variability measures and tested which of them, if any, would best predict performance in metacontrol-sensitive tasks.

We used two tasks in which high performance requires cognitive persistence (i.e., the Stroop task and the Remote Associates Task (RAT)), and a task in which high performance depends on cognitive flexibility (i.e., the Alternate Uses Task (AUT)). Given that metacontrol biases cannot (yet) be assessed directly, we followed the previous experimental logic of comparing individual differences in tasks that rely (more) on persistence with tasks that rely (more) on flexibility^26^. Persistence is assumed to lead to a strong focus on the present goal and information strictly related to that goal, which suggests that a high degree of persistence would lead to better performance in tasks that require a strong focus on some stimuli and neglect of others. The Stroop task^35^ is an excellent example for such a task. In the classical Stroop task, participants are to respond to the color of colored words while ignoring the word meaning (e.g., responding “green” to the word “RED” written in green ink^35–37^). To be successful in this task, one has to process task-relevant information (i.e., color “green”) and ignore task-irrelevant information (i.e., word “RED”). Individuals usually respond slower in incongruent trials (in which the color of the word and meaning are different) than in congruent trials (in which the color of the word and meaning are same), which is known as the Stroop effect. A smaller Stroop effect can be taken to indicate a better ability in reducing cognitive conflict, which is supposed to benefit from a metacontrol bias towards persistence (e.g., Dreisbach & Goschke, 2004, who applied this logic to similar tasks^38^). In comparison, a larger Stroop effect implies a stronger impact from task-irrelevant information, which indicates a metacontrol bias towards flexibility. As some researchers argue that reaction time (RT) difference scores are sometimes unreliable in individual differences research^39^, we also considered intra-individual variability (IIV) of Stroop performance, which can be taken to reflect the stability of metacontrol over time. More trial-to-trial variability which was potentially induced by more frequent strategy readjustments, would indicate lesser stability of metacontrol states, i.e., higher flexibility. Conversely, less trial-to-trial variability in Stroop performance would indicate more persistence.

A second persistence-heavy task we considered was the Remote Associates Task (RAT). RAT is typically used to measure convergent thinking, which is one aspect or component of human creativity^40^. It requires participants to find a single solution under highly constrained search conditions: they are presented with three words and are requested to specify the one word that can be combined with either of them (e.g., “Market”, “Glue”, and “Man”, with the solution “Super”). While this task does require a certain degree of flexibility (in repeatedly searching through memory and considering novel possible targets), its reliance on persistence is much stronger than in tasks testing divergent thinking^26,41^. Accordingly, participants with comparably better performance in the RAT would be considered to have a stronger bias towards persistence than participants with worse performance^42^.

As a flexibility-heavy task, we employed the Alternate Uses Task (AUT)^43,44^. This task is traditionally used to assess divergent thinking, another component of human of creativity, requiring to generate new ideas and to overcome more familiar, but currently misleading ideas^43,44^. As an example, a participant might be presented with the label or picture of a brick and asked to report all kinds of uses that a brick might have, including very uncommon ones. The AUT does need some degree of persistence (in keeping the original concept active to check it for possible uses) but it relies much more on flexibility^26,41^. Accordingly, participants with comparably better performance in the AUT would be considered to have a stronger bias towards flexibility than participants with worse performance^42^.

In sum, the present study explored whether and how resting-state BOLD signal variability is associated with inter-individual differences in metacontrol biases towards persistence or flexibility. We examined different indicators of brain variability and three different tasks drawing on cognitive persistence or flexibility. Our main question was whether two indicators are significantly related to performance in the three behavioral tasks and whether these associations would differ between tasks tapping into persistence biases and tasks tapping into flexibility biases. We were also interested in possible differences between the two indicators in the way they are associated with such behavioral differences but had no specific hypothesis regarding such differences.

## Results

### Behavioral findings

**Figure 1:**
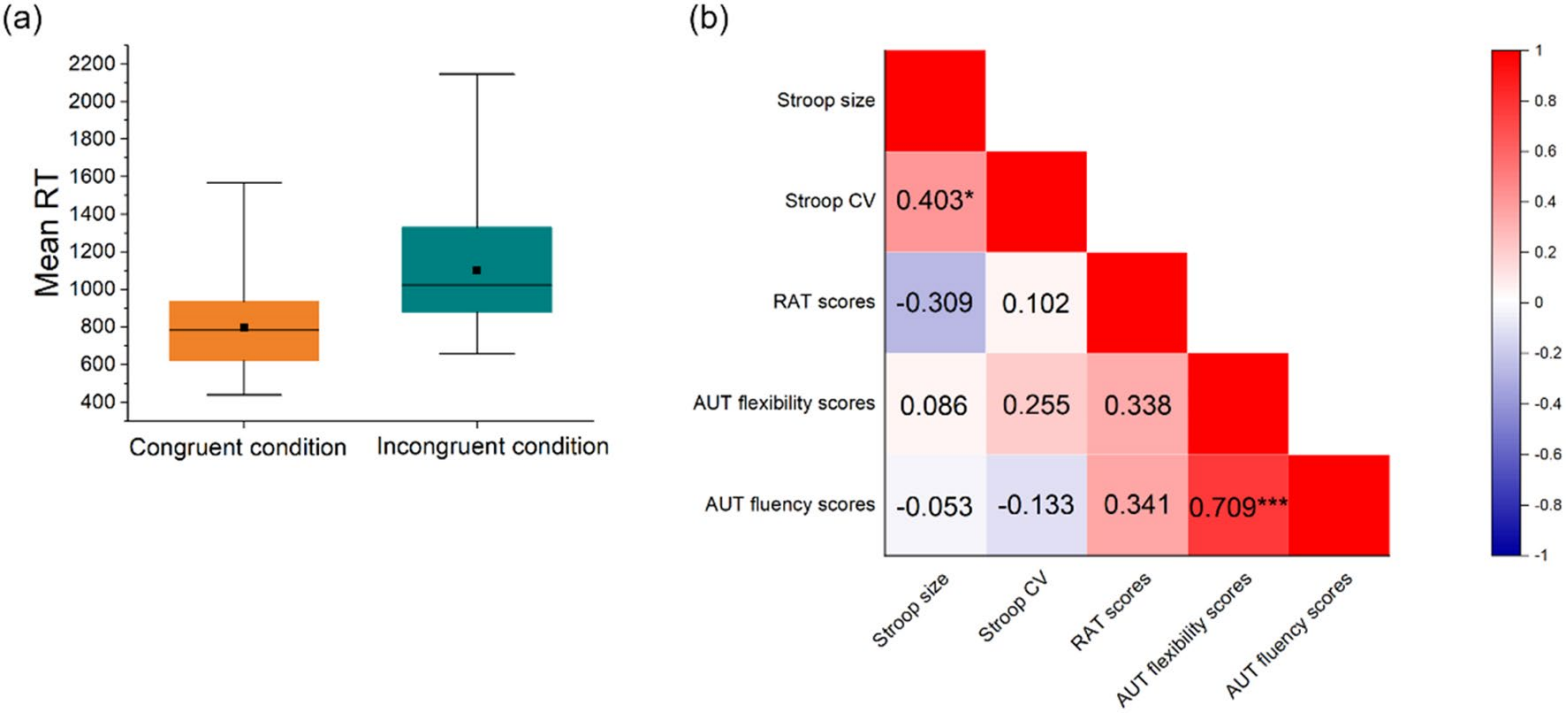
Statistics of mean RT in the Stroop task and inter-correlations between behavioral assessments. (**a**) Mean reaction time (RT) in (corresponding) incongruent condition was larger than RT in (corresponding) congruent condition; (**b**) inter-correlation between the size of Stroop effect, RT-CV of Stroop task, RAT scores, AUT flexibility scores and AUT fluency scores. *p < 0.05, ***p < 0.001.

The analysis of the Stroop data (*n* = 32) yielded a standard Stroop effect, with longer mean RTs in incongruent trials (1100 ms, *SD* = 317 ms) than in congruent trials (797 ms, *SD* = 234 ms), *t*(31) = 4.34, *p* < 0.001, *d* = 1.09) (see Fig. 1a). Performance accuracy and speed were not significantly correlated (congruent trials: *r* = 0.181, *p* = 0.323; incongruent trials: *r* = 0.260, *p* = 0.151), which rules out a speed–accuracy trade-off. Intra-individual variability of Stroop performance (RT-CV) was 0.315 ± 0.062 ms. In the RAT, participants solved 6.22 items correctly on average (*SD* = 4.09). In the AUT, inter-rater reliability was assessed by intraclass correlation coefficients (ICC), which were moderate for flexibility scores (ICC _shoe_ = 0.571, ICC _stone_ = 0.650) and for fluency scores (ICC _shoe_ = 0.705, ICC _stone_ = 0.665). The averaged AUT flexibility scores from both raters were 7.50 ± 2.04, and the averaged AUT fluency scores were 9.78 ± 2.26. Histograms displaying the distribution of above-mentioned variables are provided in the supplementary Fig. S3.

In order to test whether metacontrol-bias parameters extracted from various tasks and different measures were related, we applied an inter-correlation analysis between the size of Stroop effect, RT-CV of Stroop task, RAT scores, AUT flexibility scores, and AUT fluency scores. As displayed in Fig. 1b, the size of Stroop effect was significantly positively correlated with RT-CV (*r* = 0.403, *p* = 0.022). A highly positive correlation was also found between AUT flexibility scores and AUT fluency scores (*r* = 0.709, *p* < 0.001). Correlations between other measures were not significant. These results may indicate that participants are biased towards persistence or flexibility to a different extent, depending on the task demands.

### Resting-state independent components findings

**Figure 2:**
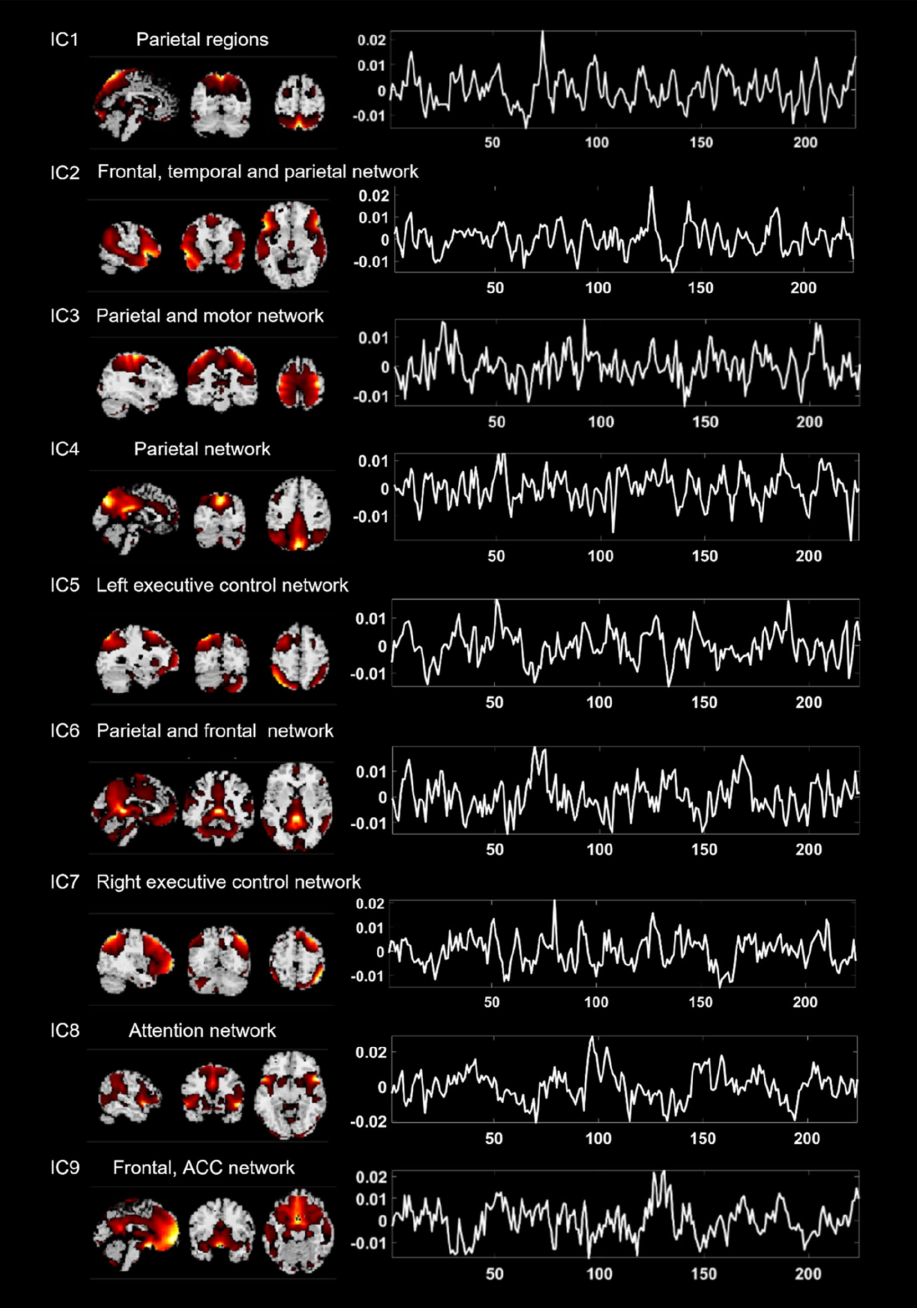
Spatial maps (*Z*-threshold > 1.0, in the left panel) and time series (in the right panel) for selected independent components of the mean for all participants.

The spatial maps at the threshold of *Z* > 1.0 and time courses of our selected ICs are shown in Fig. 2. IC1 and IC4 mainly reflect activities in bilateral precuneus, superior and inferior parietal regions, within the parietal cortex. IC2 includes bilateral inferior prefrontal gyrus, middle temporal gyrus, and angular gyrus. Bilateral inferior parietal regions, postcentral and precentral areas are involved in IC3, which was defined as a parietal and motor network. IC5 reflects the left-sided executive control network, including the left prefrontal and parietal cortex, while IC7 represents the right executive control network^45^. IC6 mainly includes the bilateral middle part of orbital frontal gyrus and precuneus which was defined as the frontal and parietal network. IC8 represents activity in the anterior cingulate cortex, the prefrontal cortex, and the bilateral insular, which were denoted as the attention network^46^. IC9 mainly reflects activity in the prefrontal cortex and extends to the anterior cingulate cortex.

To assess whether brain variability correlated between different measures, we tested the Pearson correlations between brain variability measured by SD and MSSD. Results showed that SD and MSSD of BOLD signals were highly positively correlated for all ICs (see Table 1 for details), suggesting that these two approaches are consistent in assessing the temporal variability of rsfMRI data.

**Table 1:** Pearson correlations between brain variability measured by SD and MSSD.

ICs	Correlation between SD and MSSD	
	r	p
IC1	0.742	< 0.0001
IC2	0.551	0.0011
IC3	0.829	< 0.0001
IC4	0.731	< 0.0001
IC5	0.629	0.0001
IC6	0.710	< 0.0001
IC7	0.623	0.0001
IC8	0.523	0.0021
IC9	0.586	0.0004

*IC* independent component, *SD* standard deviation, *MSSD* mean squared successive difference.

### Resting-state BOLD variability and individual difference in metacontrol policies

**Figure 3:**
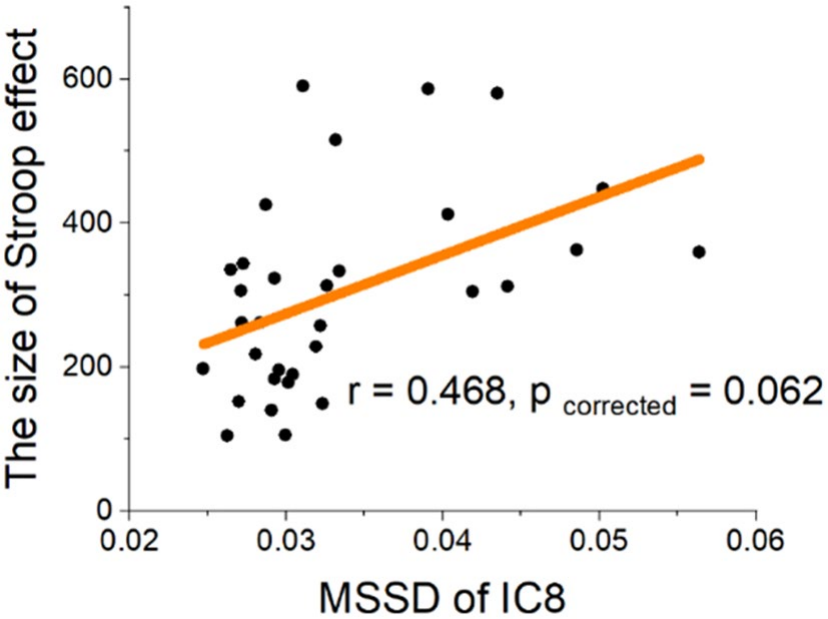
The correlation between the size of Stroop effect and brain variability of the attention network (i.e., IC8) was close to significance. The higher the brain variability of IC8 estimated by MSSD, the larger the size of Stroop effect.

The analysis of the SD measure revealed that the SD of all selected components was positively correlated with the size of the Stroop effect. A pattern of positive correlations was also obtained between MSSD of all components and the size of Stroop effect. A close to significance positive correlation was found between MSSD of IC8 (i.e., attention network) and the size of Stroop effect (*r* = 0.468, *p*_uncorrected_ = 0.007, *p*_corrected_ = 0.062) (Fig. 3; see Table 2 for details). We performed a supplementary analysis in which we include two participants who were excluded due to the extreme value in the Stroop effect. Results showed that the association between MSSD of IC8 and Stroop effect size is not significant (see the supplementary Fig. S4 for an updated scatterplot). No significant correlations were found between RT-CV of Stroop task and brain variability as measured by SD, or MSSD.

**Table 2:** Correlations between brain variability measured by SD, MSSD, and metacontrol policies measured by the size of Stroop effect, IIV of Stroop performance (RT-CV), RAT scores, AUT flexibility scores, and AUT fluency scores.

Brain variability measures	ICs	Size of Stroop effect			RT-CV of Stroop task			RAT scores			AUT flexibility scores			AUT fluency scores		
		r	p_uncorrected_	p_corrected_	r	p_uncorrected_	p_corrected_	r	p_uncorrected_	p_corrected_	r	p_uncorrected_	p_corrected_	r	p_uncorrected_	p_corrected_
SD	IC1	0.117	0.523	1.000	0.030	0.870	1.000	− 0.117	0.522	1.000	0.024	0.898	1.000	0.089	0.627	1.000
	IC2	0.260	0.150	1.000	0.003	0.986	1.000	− 0.305	0.089	0.805	0.135	0.461	1.000	0.141	0.441	1.000
	IC3	0.379	0.032	0.292	0.123	0.502	1.000	− **0.505**	**0.003**	**0.029**	− 0.086	0.639	1.000	− 0.054	0.768	1.000
	IC4	0.422	0.016	0.145	0.175	0.337	1.000	− 0.146	0.424	1.000	0.027	0.883	1.000	0.014	0.937	1.000
	IC5	0.258	0.154	1.000	− 0.009	0.962	1.000	− 0.223	0.221	1.000	0.071	0.701	1.000	0.226	0.213	1.000
	IC6	0.319	0.076	0.680	− 0.128	0.487	1.000	− **0.508**	**0.003**	**0.027**	− 0.187	0.307	1.000	− 0.063	0.730	1.000
	IC7	0.116	0.529	1.000	0.000	0.998	1.000	− 0.173	0.343	1.000	0.092	0.617	1.000	0.125	0.495	1.000
	IC8	0.339	0.057	0.517	0.215	0.238	1.000	− 0.194	0.286	1.000	0.216	0.236	1.000	0.030	0.870	1.000
	IC9	0.334	0.062	0.554	0.286	0.113	1.000	− 0.440	0.012	0.105	0.034	0.852	1.000	− 0.006	0.974	1.000
MSSD	IC1	0.325	0.070	0.629	− 0.005	0.978	1.000	− 0.321	0.074	0.663	− 0.108	0.558	1.000	0.031	0.864	1.000
	IC2	0.406	0.021	0.192	− 0.023	0.902	1.000	− 0.376	0.034	0.306	− 0.019	0.918	1.000	0.160	0.380	1.000
	IC3	0.426	0.015	0.135	0.074	0.686	1.000	− **0.470**	**0.007**	**0.059**	− 0.141	0.443	1.000	− 0.168	0.357	1.000
	IC4	0.315	0.079	0.710	0.024	0.896	1.000	− 0.178	0.330	1.000	− 0.217	0.233	1.000	0.022	0.907	1.000
	IC5	0.397	0.024	0.220	0.044	0.811	1.000	− 0.279	0.122	1.000	0.038	0.837	1.000	0.115	0.531	1.000
	IC6	0.346	0.052	0.470	− 0.096	0.603	1.000	− **0.543**	**0.001**	**0.012**	− 0.128	0.486	1.000	0.040	0.827	1.000
	IC7	0.344	0.054	0.487	0.016	0.929	1.000	− 0.204	0.263	1.000	0.039	0.831	1.000	0.193	0.289	1.000
	IC8	**0.468**	**0.007**	**0.062**	0.233	0.200	1.000	− 0.365	0.040	0.358	− 0.070	0.702	1.000	− 0.084	0.649	1.000
	IC9	0.433	0.013	0.119	0.046	0.801	1.000	− **0.510**	**0.003**	**0.026**	− 0.066	0.720	1.000	0.005	0.979	1.000

Spearman correlation was used for RAT scores, AUT flexibility scores and AUT fluency scores.

*IC* independent component, *SD* standard deviation, *MSSD* mean squared successive difference, *RT-CV* coefficient of variation in reaction time, *RAT* remote associates task, *AUT* alternate uses task, *P corrected* Bonferroni corrected p value.

Significant values are in bold.

**Figure 4:**
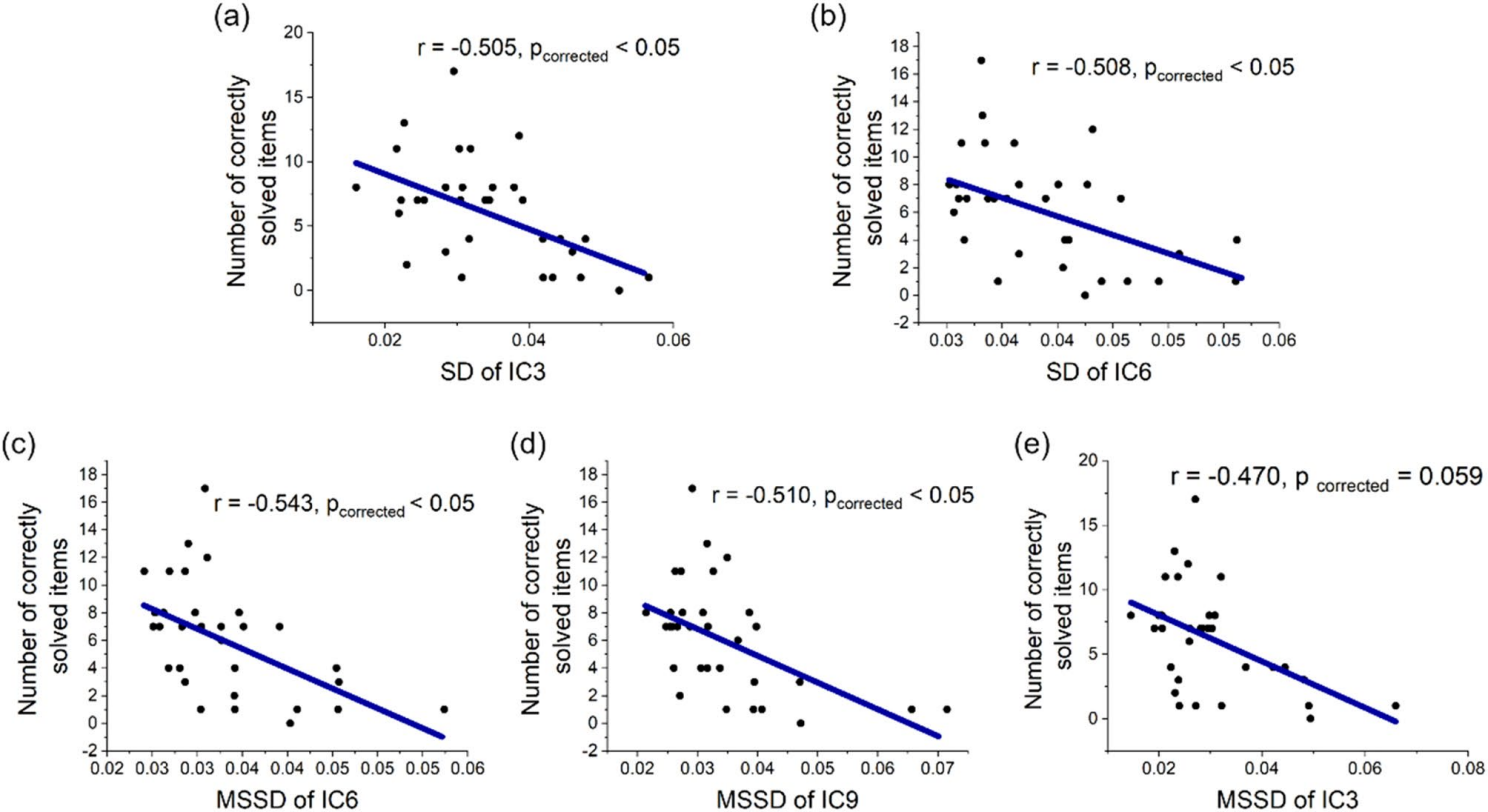
RAT performance was significantly (or, in the case of e, close to significantly) negatively correlated with brain variability of the parietal and motor network (i.e., IC3), parietal and frontal network (i.e., IC6), frontal and ACC network (i.e., IC9). Brain variability was calculated using SD in (**a**,**b**); brain variability was measured by MSSD in (**c**–**e**).

Regarding RAT performance, the SD of all ICs revealed negative correlations. SD of IC3 (i.e., parietal and motor network) and IC6 (i.e., parietal and frontal network) was significantly negatively correlated with RAT performance (IC3: *r* = − 0.505, *p*_uncorrected_ = 0.003, *p*_corrected_ < 0.05; IC6: *r* = − 0.508, *p*_uncorrected_ = 0.003, *p*_corrected_ < 0.05) (see Fig. 4a,b). A similar pattern of negative correlations was displayed between MSSD of all components and RAT scores. Most significant negative correlations were found between MSSD of IC6 (i.e., parietal and frontal network), IC9 (i.e., frontal and ACC network) and RAT performance (IC6: *r* = − 0.543, *p*_uncorrected_ = 0.001, *p*_corrected_ < 0.05; IC9: *r* = − 0.510, *p*_uncorrected_ = 0.003, *p*_corrected_ < 0.05) (see Fig. 4c,d). We found a close to significant negative correlation between MSSD of IC3 and RAT scores (*r* = − 0.470, *p*_uncorrected_ = 0.007, *p*_corrected_ = 0.059) (see Fig. 4e). These results were replicated in the supplementary analysis in which two excluded participants were included (see supplementary Fig. S5 for details).

AUT flexibility and fluency scores were not significantly related to brain variability.

### Resting-state BOLD signal variability and metacontrol in the extended dataset

With 69 participants, we obtained 11 ICs which reflect activities in control-related brain networks (see the supplementary Fig. S7 for details). The temporal variability measured by SD and MSSD was highly positively correlated (see supplementary Table S1). SD and MSSD of all selected ICs revealed negative correlations with the RAT score. We found a significant negative correlation between the MSSD of frontal motor regions (i.e., new IC3, see Fig. S7 for details) and the RAT score (*r* = -0.350, *p*_uncorrected_ = 0.003, *p*_corrected_ < 0.05) (see Fig. S8 and Table S2). No significant association was detected between brain variability of other ICs and RAT scores. AUT flexibility scores and AUT fluency scores were not significantly correlated with SD or MSSD of selected ICs.

## Discussion

The present study explored the relationship between the individual’s resting-state BOLD signal variability and individual differences in metacontrol biases towards persistence or flexibility. Two BOLD signal variability measures were compared. We found that resting-state BOLD signal variability measured by SD and MSSD was highly positively correlated. Notably, our results suggest that higher levels of resting-state BOLD variability measured by MSSD in the attention network, parietal and frontal network, frontal and ACC network, parietal and motor network, and variability measured by SD in the parietal and motor network, parietal and frontal network were associated with lesser persistence (or more flexibility) (denoted by larger Stroop effect or worse RAT performance) than lower levels of brain variability in these networks.

Correlations between two brain variability measures suggest that resting-state BOLD signal variability estimated by SD and MSSD is highly correlated. The high correlation between the SD measure and MSSD measure is consistent with findings from Garrett and colleagues^7^. Although SD as a measure of brain variability has been criticized for its dependence on shifts in the mean and MSSD was recommended to prevent this problem, our findings where MSSD and SD show highly consistent results suggest that SD is an appropriate variability measure in resting-state fMRI data where (mean) signals are relatively constant. We found that brain signal variability measured by SD and MSSD in a range of resting-state networks was positively associated with metacontrol biases towards flexibility but negatively associated with metacontrol biases towards persistence. Our findings extend previous knowledge of the relationship between brain variability and human behavior in two ways:

First, resting-state BOLD signal variability is meaningful and can tentatively be taken as a neural marker of metacontrol biases towards persistence or flexibility. Previous investigations have identified the *on-task* brain variability, which varies between cognitive demands^6^, attentional states^15^, task conditions^47^, and perceptual input^48^. We suggest that *off-task* variability can also be used as a trait-like neural marker of the individual metacontrol bias and, thus, as a predictor of individual cognitive control performance.

Second, although numerous studies demonstrate general positive effects of higher brain variability on cognitive performance^4–7,13,48,49^, our results suggest that the beneficial effect of brain variability may depend on cognitive demands and metacontrol states involved. Our findings are in line with the previous task-based fMRI study suggesting that higher brain signal variability levels are beneficial for task switching but detrimental for distractor inhibition^16^. Hence, brain variability should not be considered as a general performance booster, but as a factor that can be beneficial for some tasks but impair performance in others. How might signal variability in the brain translate into metacontrol biases towards persistence or flexibility? Researchers have proposed that dopamine (DA) and inter-individual differences in DA levels and/or the dynamics of these levels over time are promising candidates for linking characteristics of neural processing, like differences in neural variability, to behavior^5,50–52^ and some evidence suggests that dopaminergic (or catecholamine system activity) is associated with metacontrol^53–56^.

According to the computational model proposed by Durstewitz and Seamans^57^, D2-dominated state related to a low energy barrier among activity states would allow easier and faster transition between different cortical network states^16^. This D2-dominated state facilitates switching among representations at the behavioral level and supports metacontrol biases towards flexibility^26,57^. Conversely, D1-dominated states are associated with a high energy barrier leading to more stable brain activity patterns and a more difficult transition between different network states^16,57^.

At the same time, this D1-dominated state boosts the robustness of items in working memory and promotes metacontrol biases towards persistence^26,57^. Evidence from simulation research suggests that dynamics of the brain's intrinsic properties may help keep the system in a state where different subnetworks compete with each other^28^. Such an active resting-state (at an optimal level) can be sensitive to external signals, which can trigger brain activity during different tasks, thus supporting behavioral exploring and switching. In contrast, sensitivity to external stimuli makes people more likely to be distracted by task-irrelevant stimuli.

We found that resting-state BOLD variability of parietal and motor network (IC3), parietal and frontal network (IC6), attention network (IC8), frontal and ACC network (IC9) was positively associated with metacontrol biases towards flexibility but negatively associated with metacontrol biases towards persistence. Previous work suggests that distractor inhibition and task switching rely on a shared frontoparietal network, and brain activity varies depending on the exact cognitive processing involved^58^. As a control network, the frontoparietal network plays a crucial role in task adaptation, implementation and flexible modulation of cognitive control^59^. Moreover, the frontoparietal network is a globally functional hub that flexibly interacts with other brain networks. Higher variability in frontal and parietal regions may indicate more dynamic connectivity between brain networks with the frontoparietal network as the hub, and thus supports the flexibility of metacontrol, but hamper persistence of metacontrol^60,61^. The attention network which mainly includes ACC, prefrontal cortex and insular has been shown to be involved in sustained focus on task-relevant information and conflict resolution^62,63^. A variable attention network may reveal flexible attention resources allocation, which is beneficial for flexibility but detrimental for persistence.

Whereas the analyses of the Stroop and the RAT data provide a rather consistent picture, this is not the case with respect to the AUT findings. On the one hand, previous studies have rarely found RAT performance to be an exact mirror image of AUT performance; rather, various manipulations affected either only one of the two tasks or at least one of the more than the other^64,65^. This suggests that both tasks are likely to capture aspects of metacontrol persistence and flexibility, but they can hardly be viewed as a direct measure of the respective metacontrol states. It is also likely that they differ in sensitivity, presumably depending on the experimental setting. Hence, it does not seem to be odd per se that only one of the two creativity tasks showed systematic effects. On the other hand, however, it is also possible that our particular assessment of divergent thinking was suboptimal. Due to the time limit in Qualtrics, our AUT task only allowed up to 6 responses within a short time duration for each item. This might have created ceiling effects, so that especially the fluency and flexibility scores were likely to be less sensitive to interindividual differences than the standard versions of the AUT. This must have reduced the variability of the data, which in turn could have worked against finding significant correlations. Accordingly, we are reluctant to draw strong conclusions from the absence of correlations related to the AUT.

Another potential limitation of our explorative study is the sample size, which in turn resulted from our use of already collected data. Larger sample sizes would be beneficial for probing brain-behavior relationships. Accordingly, we consider the outcomes of the present study as preliminary and in need of replication, but at the same time encouraging for further studies on the relationship between brain variability and metacontrol policies.

To conclude, we aimed to explore the relationship between resting-state BOLD signal variability and metacontrol policies and compared two previously used brain variability estimation metrics. We demonstrated that temporal brain variability during resting-state is associated with metacontrol biases towards persistence or flexibility, highlighting the importance of temporal variability of brain activity in understanding the neural underpinnings of cognitive control. Moreover, we found that BOLD signal variability is antagonistically related to metacontrol biases towards persistence or flexibility, suggesting that the beneficial effect of brain variability on cognitive control may depend on the metacontrol modes involved. At last, the SD and MSSD indices of rsfMRI brain variability provide consistent pictures for predicting behavioral cognitive control.

## Materials and methods

### Participants

Our sample consisted of thirty-two right-handed adults (21 females; age 18–35 years; *M* = 23.81, *SD* = 3.53). The raw dataset, which has been reported in a previous study^66^, included 40 university students reporting no history of psychiatric or neurological disorders. Six participants were excluded because of missing data for the Stroop task, RAT or AUT, or resting-state fMRI scanning; two participants were excluded because of extremely large or small Stroop effect size (i.e., exceeding group mean ± 2 standard deviations). The mean framewise displacement (FD) of all remaining participants was smaller than 0.5 mm. The present study was approved by the Psychology Research Ethics Committee of Leiden University. The original study was approved by the Internal Review Board of the Erasmus Research Institute of Management, and all participants provided written informed consent for their participation. The current study and original study were conducted in accordance with the Declaration of Helsinki.

### Behavioral assessment

#### Color-word matching Stroop task

**Figure 5:**
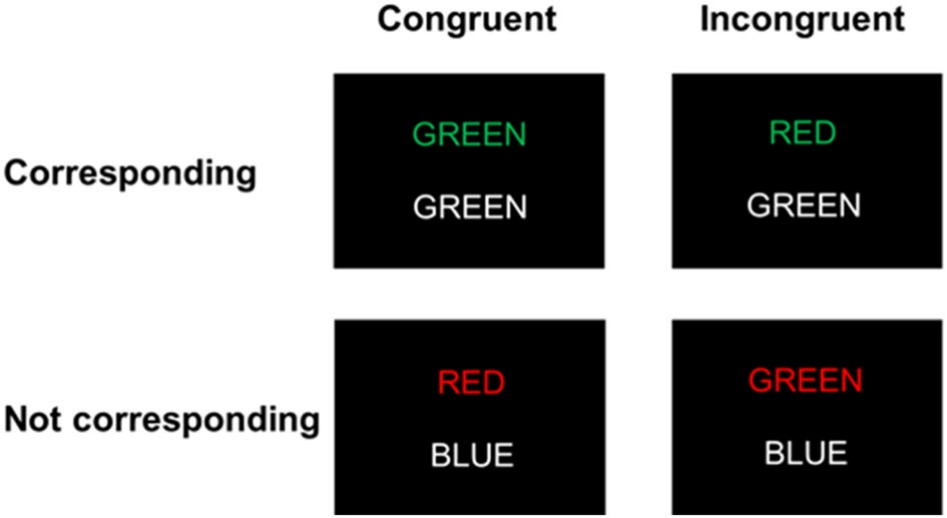
Examples for conditions and design of the color-word matching Stroop task. For the upper two examples, the correct answer would be “YES,” for the lower two examples, the correct answer would be “NO.”

An adapted version of the Stroop task^35^ was used. In this task, two rows of letters appeared on screen, and participants were instructed to decide as quickly as possible whether the color of the top row letters correspond to the color name written at the bottom row by pressing one of two buttons (see Fig. 5). In congruent trials, the top row consisted of a color word (“RED,” “GREEN,” “BLUE,” or “YELLOW”) printed in a color that matches its semantic meaning (e.g., “RED” presented in red ink), and the bottom row consisted of a color word printed in white ink. For incongruent trials, the color word in the top row printed in a color that mismatches its semantic meaning (e.g., “RED” presented in green ink). The bottom row letters were identical to the congruent condition. Participants performed 72 trials in the MRI scanner, containing 36 congruent trials and 36 incongruent trials. In half of the trials, the color of top row word corresponded to bottom color word (corresponding trials), while the color of top row word not corresponded to bottom word in the other half (not corresponding trials).

Each trial started with a fixation period of 2000–4000 ms, followed by the stimuli presented for a maximum time of 3000 ms. Afterwards feedback appeared for 1000 ms. To prevent participants from focusing on the bottom word and not attending the word in the top row, the top-row word was presented 100 ms before the bottom word. If no response was given within 3000 ms from the onset of the stimulus presentation, an incorrect response was registered.

We calculated two parameters from the Stroop task as estimations of metacontrol biases: first, the size of Stroop effect (mean RT for incongruent trials minus mean RT for congruent trials). As we mentioned before, a smaller Stroop effect indicates bias towards persistence, while a larger Stroop effect indicates bias towards flexibility. Note that in our word-matching version of the Stroop task, the size of the Stroop effect may depend on the type of answer (yes or no), i.e., on the color-word correspondence^67^. Specifically, in non-corresponding trials (when the answer was ‘NO’), the conflict generated by the Stroop effect may facilitate a ‘no response’, which may work against the Stroop effect. Hence, a standard Stroop effect may only occur with correspondence (when the answer was ‘YES’). Therefore, we calculated the Stroop effect by subtracting the mean RT for corresponding congruent trials from the mean RT for corresponding incongruent trials. As RT difference scores are sometimes unreliable in assessing individual differences^39^, we also calculated the intra-individual variability (IIV) of Stroop RT as a second metacontrol measure. The IIV of Stroop RT was estimated by the RT coefficient of variation across all trials (RT-CV: SD divided by mean). Greater RT-CV would reflect lesser stability of metacontrol states in the Stroop task, i.e., a bias towards flexibility. In contrast, smaller RT-CV would be taken as a bias towards metacontrol persistence. The mean accuracy across all trials was 0.90 (*SD* = 0.07) (see the supplementary Fig. S1 for the histogram). The Stroop effect and RT-CV were calculated on correct trials only. The response latency in each trial ranged from 344 to 2995 ms.

#### Remote associates task (RAT)

In each trial of this task, participants were to find a single word that can be combined with each of the three presented stimulus words (e.g., cottage, swiss, cake = “cheese”)^40^. Participants had to complete 17 trials within 5 min. This task was completed via Qualtrics outside the scanner. To complete the RAT, participants were assumed to engage in convergent thinking, which was assumed to rely on a persistence bias^68^.

#### Alternate uses task (AUT)

Participants were presented with two everyday objects (i.e., shoe, stone) and asked to name as many possible uses (up to 6 uses) for each object as they can. This task was completed via the Qualtrics outside the scanner and participants had 3 min for both objects together. Performance on AUT was scored by two independent raters from four dimensions: flexibility (number of ideas in different categories), fluency (number of uses one can think of), originality (uniqueness of responses), and elaboration (the level of details in responses). As flexibility and fluency require switching between different ideas and considering multiple solutions^44^, we used flexibility scores and fluency scores which were averaged between two raters as metacontrol biases measures. Higher scores indicated more tendency towards flexibility, while lower scores indicated more tendency towards persistence^44^.

### MRI data acquisition

MRI scanning was performed on a 3 T Siemens Verio MRI system. Resting-state functional data were acquired by a T2*-weighted gradient-echo, echo-planar pulse sequence in descending interleaved order (repetition time (TR) = 2030 ms, echo time (TE) = 30 ms, flip angle = 75°, slice thickness = 3.0 mm, in-plane resolution = 3.0 × 3.0 mm, 64 × 64 voxels per slice). In addition to functional imaging, a T1-weighted image was acquired at the resolution of 1.0 × 0.5 × 0.5 mm for anatomical reference (192 sagittal slices, TR = 1900 ms, TE = 2.26 ms, flip angle = 9°).

#### Resting-state functional data preprocessing

Data preprocessing was performed using DPASF (http://rfmri.org/DPARSF), a Matlab toolbox for resting-state fMRI data processing and analysis^69,70^. The first 10 volumes were discarded, and then slice-time correction and realignment were performed. Head motion was assessed by frame-wise displacement (FD)^71^. All participants’ mean FD were smaller than 0.5 mm. Individual T1-weighted images were co-registered to the mean functional image and then segmented into gray matter, white matter (WM), and cerebrospinal fluid (CSF). Transformations from individual native space to MNI space were computed with the DARTEL tool^72^, and then the functional images were normalized to MNI space with warped parameters. Lastly, all functional images were smoothed with a 6 mm full width at half maximum (FWHM) Gaussian kernel.

#### Group independent component analysis

As previous studies note that brain signal variability is region-specific^16,47^, we only selected control-related networks (i.e., independent components) which were obtained from the independent component analysis (ICA). ICA was performed using the GIFT Toolbox (https://www.nitrc.org/projects/gift) to identify temporally coherent networks which are spatially distinct. Following the processing protocol used in the previous study^73^, pre-processed functional images were firstly intensity-normalized. Subsequently, each participant’s data were reduced to 70 principal components. Then, group-level decomposition was performed using the Infomax algorithm^74^, which resulted in 25 spatially independent components (ICs) and associated time courses. To improve the reliability of IC-decomposition, the Infomax ICA algorithm was repeated 20 times using the ICASSO toolbox^75^. Afterward, the obtained 25 ICs were visually inspected to exclude noise components. We then compared all non-noise components’ spatial topology to the pre-defined resting-state network templates^45,46^. The ICs reflecting activities in the executive control network, attention network, prefrontal, and parietal regions were identified and used for further analyses. Participant-specific spatial maps and time courses were then estimated using the dual regression back-reconstruction method^76^. We did not further scale the components due to the preprocessing step of intensity normalization, which returns back-reconstructed maps in units of percent signal change. Spatial maps for excluded components are shown in the supplementary Fig. S2.

#### Resting-state BOLD signal variability calculation

We estimated resting-state BOLD signal variability using component-wise within-participant measures. For each component and each participant, BOLD variability was calculated. Here, we used two brain signal variability measures listed below.

First, we calculated the standard deviation (SD) of BOLD signals for each component and each participant.

As a second measure, we estimated the variability of time courses in selected ICs via mean squared successive difference (MSSD)^30,31^. As a non-biased estimation to SD, MSSD reflects moment-to-moment BOLD signal variability that is less sensitive to low-frequency drift^77^ and independent from shifts in the mean^7^. For each IC and each participant, we subtracted BOLD signals in time point t from time point t + 1, and then squared the average of all subtractions across the entire time series. (Eq. (1): t and t + 1 are two successive time points belonging to the same component time course, n is the number of time points in each component).

1 $$MSSD= \sqrt{\frac{{\sum }_{t = 1}^{n - 1}{{(x}_{t +1}- {x}_{t})}^{2}}{n- 1}}$$

### Statistical analysis

To examine the relationship between resting-state BOLD signal variability and individual differences in metacontrol policies, we correlated the size of Stroop effect, Stroop RT-CV, RAT scores, AUT flexibility scores, and AUT fluency scores with brain variability estimated by SD and MSSD, respectively. As nine components were included for correlation analysis, Bonferroni correction was used to control for the increased risk of a type I error. Note that the theoretical meaning of the signs/directions of the correlations varies with task scores: Whereas higher scores in the two Stroop measures and the AUT scores imply stronger bias towards flexibility (and lower scores stronger bias towards persistence), higher scores in the RAT imply stronger bias towards persistence (and lower scores stronger bias towards flexibility).

### Resting-state BOLD signal variability and metacontrol in an extended dataset

The original study^66^ from which we obtained data for the current study collected behavioral and neural data from four separate samples (two big and two small samples). Besides a big sample we reported above (referred to as Sample 1), there exists a N = 41 sample which will be referred to as Sample 2. Sample 2 consisted of a different population, and neural data was collected in a different scanner than Sample 1. Participants in Sample 2 only completed creativity tasks, and RAT was tested by different items from those in Sample 1 (Detailed information can be found in the Supplementary Material S1). To test the stability of the brain-behavior correlation, we replicated the analysis of the association between resting-state brain variability and RAT performance, AUT flexibility, and AUT fluency, respectively in an extended sample consisting of both Sample 1 and Sample 2 (see the Supplementary Material S1 for details).

### Ethical approval

Our study/analytical design and our hypotheses were developed and submitted for ethical approval after the data collection was completed but before we had access to the data.

## Supplementary Information

Supplementary Information.

## Data Availability

Data is publicly available in a repository which can be accessed by the following link: https://datarepository.eur.nl/articles/dataset/Individual_differences_in_dis_honesty_are_represented_in_the_brain_s_functional_connectivity_at_rest_/17091323/1. In the present study, we used existing data initially collected for a project reported in the article *Individual differences in (dis)honesty are represented in the brain's functional connectivity at rest*^66^. Note that this study, which served other theoretical purposes, included the collection of additional data and measurements that are not reported here.
