# Supplementary material for: Resting-state BOLD signal variability is associated with individual differences in metacontrol
Source: Sci Rep. 2022 Nov 1;12:18425. doi: 10.1038/s41598-022-21703-5 (PMC9626555; doi:10.1038/s41598-022-21703-5)
Supplement: Supplementary file 1 — Supplementary Information. [file 41598_2022_21703_MOESM1_ESM.docx]

**Supplementary Information**

**Resting-state BOLD signal variability is associated with individual differences in metacontrol**

Chenyan Zhang, Christian Beste, Luisa Prochazkova, Kangcheng Wang, Sebastian P.H. Speer, Ale Smidts, Maarten A.S. Boksem & Bernhard Hommel^*^

# The distribution of accuracy on the color-word matching Stroop task


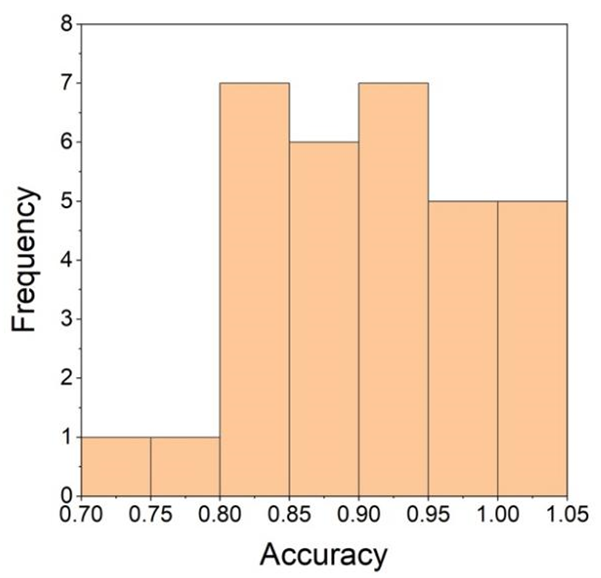


**Figure S1.** The histogram of the accuracy on the Stroop task. The accuracy was estimated based on the average of all trials in the Stroop task.

# Excluded independent components in the group independent component analysis

Spatial maps for 16 excluded independent components (ICs) were showed in Figure S2.

**
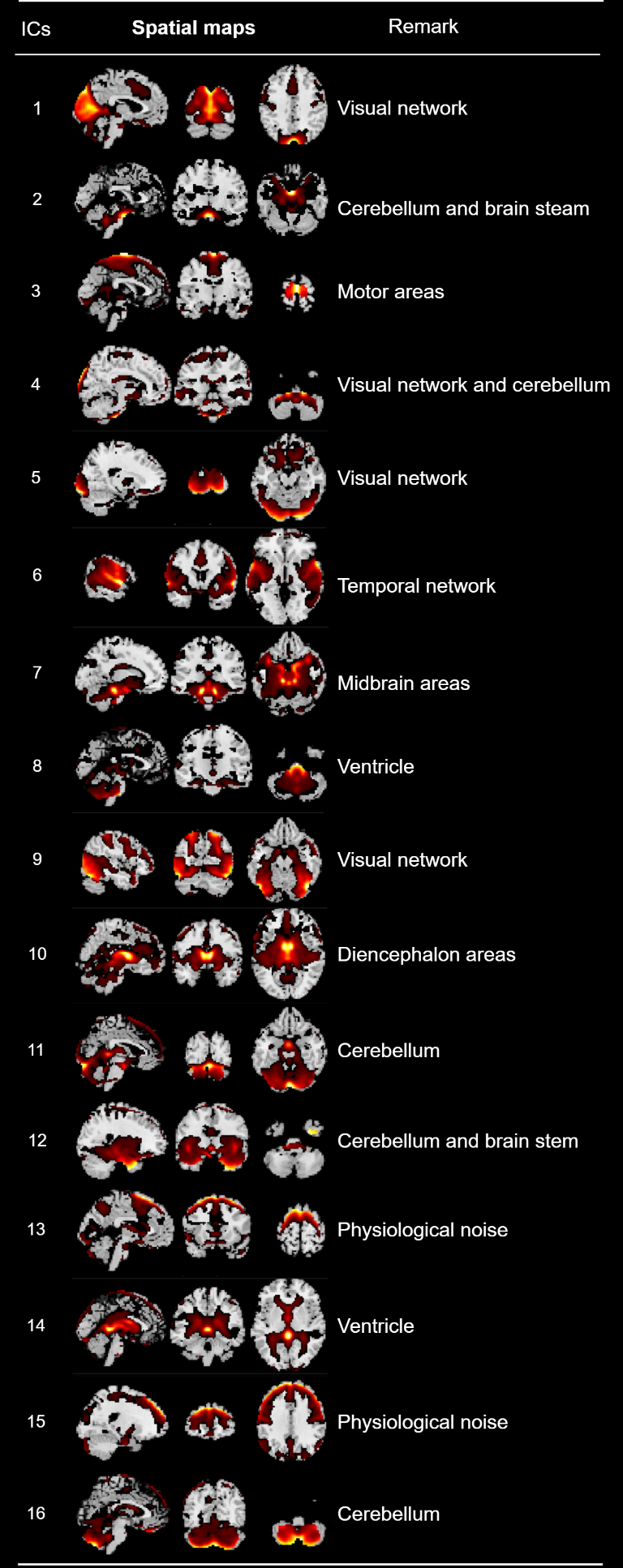
**

**Figure S2.** Spatial maps (Z-threshold > 0.1) for excluded independent components

# The distribution of RT-Stroop effect, RT-CV of Stroop performance, RAT scores, AUT fluency scores and AUT flexibility scores


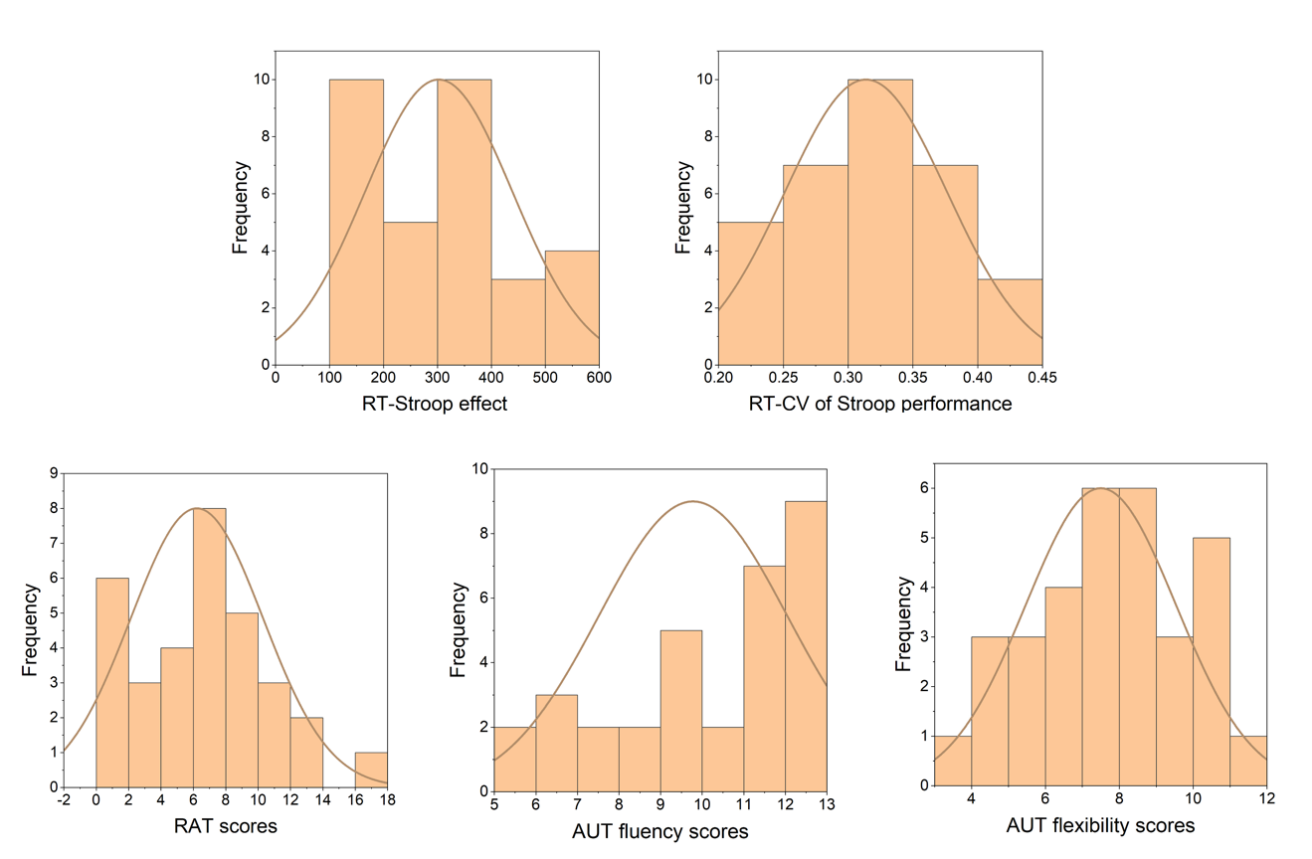


**Figure S3.** Histograms of the RT-Stroop effect, RT-CV of Stroop performance, RAT scores, AUT fluency scores, and AUT flexibility scores.

# The relationship between resting-state BOLD signal variability and metacontrol when two participants with extreme Stroop effect are included

Two participants were identified as outliers in the Stroop task. If these participants are included, we didn’t find significant correlation between BOLD signal variability and Stroop effect or Stroop RT-CV. The Figure S4 displayed an updated scatterplot of the relation between MSSD of IC8 and the size of Stroop effect.


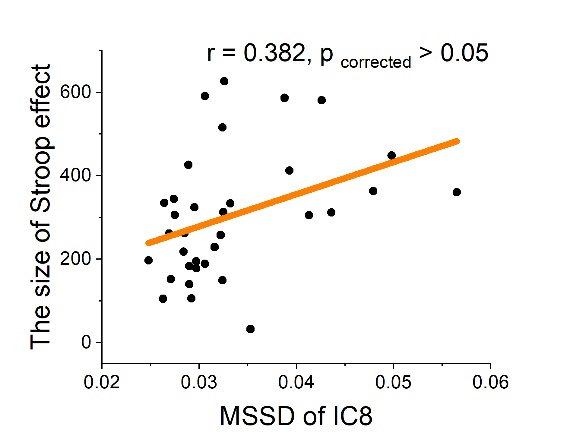


**Figure S4.** The correlation between the size of Stroop effect and brain variability of the attention network (i.e., IC8) was not significant.

The association between brain variability and the RAT performance almost remains the same (see Figure S5). More specifically, SD of IC3 (i.e., parietal and motor network) and IC6 (i.e., parietal and frontal network) was significantly negatively correlated with the RAT performance (IC3: *r* = -0.569, *p* _uncorrected_ < 0.001, *p* _corrected_ < 0.05; IC6: *r* = -0.484, *p* _uncorrected_ = 0.004, *p* _corrected_ < 0.05) (see Figure S5a and S5b). The MSSD of IC3, IC6 and IC9 was significantly negatively correlated with the RAT performance (IC3: *r* = -0.476, *p* _uncorrected_ = 0.004, *p* _corrected_ < 0.05; IC6: *r* = -0.515, *p* _uncorrected_ = 0.002, *p* _corrected_ < 0.05; IC9: *r* = -0.522, *p* _uncorrected_ = 0.002, *p* _corrected_ < 0.05) (see Figure S5c, S5d, and S5e).


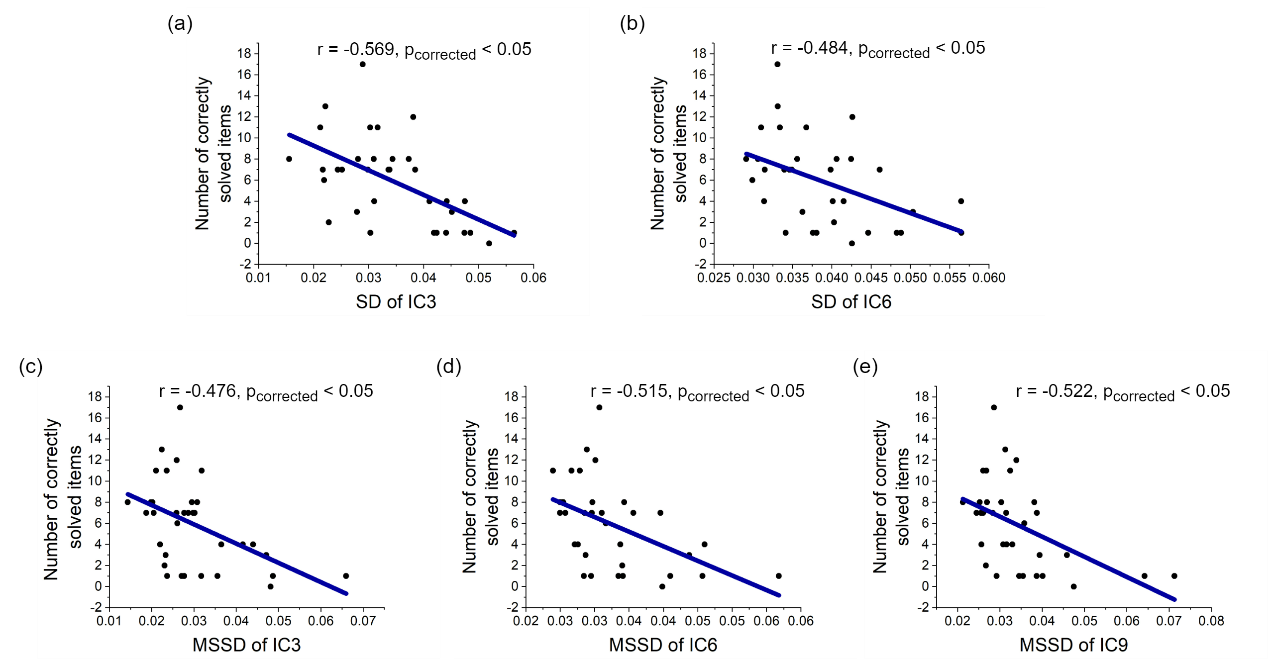


**Figure S5**. RAT performance was significantly negatively correlated with brain variability of the parietal and motor network (i.e., IC3), parietal and frontal network (i.e., IC6), frontal and ACC network (i.e., IC9). Brain variability was calculated using SD in (a) and (b); brain variability was measured by MSSD in (c), (d) and (e).

AUT flexibility and fluency scores were not significantly related to brain variability.

# Information about the Sample 2 (i.e., N = 41 sample)

## Participants

The N=41 sample consisted of a general population from a different city and neural data was collected in a different scanner than the Sample 1^1^. Four participants were excluded as they did not complete the RAT, AUT or mean FD > 0.5mm. Thirty-seven participants were remaining for further analyses (*N* = 37, 21 females; age 18 - 43 years, *M* = 24.76, *SD* = 5.63).

## Remote Associates Task (RAT)

Participants were required to complete a Dutch version of RAT^2^. RAT items in this sample were different from those in the Sample 1. Participants had to complete 17 trials within 5 minutes. This task was completed via Qualtrics outside the scanner.

## Alternate Uses Task (AUT)

Participants were asked to complete an AUT task which is similar to that reported in the main text.

## MRI data acquisition

The functional magnetic resonance images were collected on a 3T Phillips Achieva MRI system. Resting-state functional data were acquired by a T2∗-weighted gradient-echo, echo-planar pulse sequence in descending interleaved order (TR = 2000ms; TE = 27ms; flip angle = 76°; slice thickness = 3.0mm; in-plane resolution = 3.0 × 3.0 mm; 64 × 64 voxels per slice,). A T1-weighted scan was acquired using 3D fast field echo (TR = 82ms; TE = 38ms; flip angle = 8°; FOV = 240 × 188 mm; 220 slices acquired using single-shot ascending slice order and a voxel size of 1 × 1 × 1 mm). The functional scans were acquired for 8 min.

## Resting-state functional data preprocessing

The first 6 volumes were discarded to eliminate T1-equilibration artifacts from the time-series. Subsequently, preprocessing was performed using the CONN preprocessing pipeline in MATLAB. Functional images were motion-corrected using realign & unwrap procedure followed by slice-timing correction. Functional images were then co-registered to the T1 image. Both the functional and the structural data were normalized into standard MNI space. Functional data were then smoothed with a Gaussian kernel of 6 mm full width half maximum.

# Resting-state BOLD signal variability and metacontrol in the extended dataset

## Participants

The extended sample was comprised of 69 healthy adults (42 females; age 18 – 43 years; *M* = 24.32, *SD* = 4.78). 32 of them were from the Sample 1 and 37 of them were from Sample 2.

## Group independent component analysis

Preprocessed functional images from all 69 participants were entered into the GIFT toolbox for the independent component analysis. We used the same ICA analysis method as described in the main paper.

## Resting-state BOLD signal variability calculation

For each component and each participant, SD and MSSD of the BOLD signal were calculated. We then correlated the RAT score with brain variability estimated by SD, and MSSD, respectively. Bonferroni correction was used to reduce the chances of type I errors.

## Results

## Behavioral findings

In the RAT, participants solved 5.66 items correctly on average (SD = 3.31). The averaged AUT flexibility scores were 7.31 ± 1.82, and averaged AUT fluency scores were 9.64 ± 2.21. Consistent with our findings in Sample1, AUT flexibility scores and AUT fluency scores were highly positively correlated (*r* = 0.731, *p* < 0.001), while correlations between RAT scores and two AUT scores were not significant (see Figure S6).


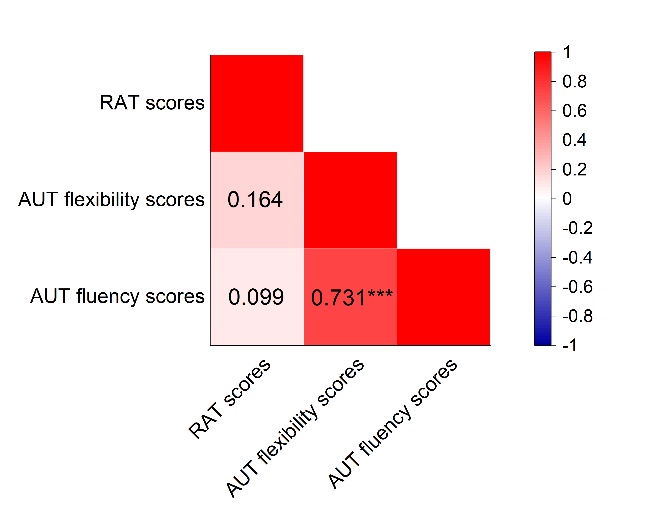


**Figure S6**. Inter-correlation between RAT scores, AUT flexibility scores and AUT fluency scores.

*Note.* * = p < 0.05, *** = p < 0.001

## Resting-state independent components findings

11 ICs which reflect the activity in the “executive control network”, the “frontal network” and the “parietal network” were chosen for the brain variability calculation. The spatial maps at the threshold of Z > 1.0 and time courses of our selected ICs were shown in Figure S7.


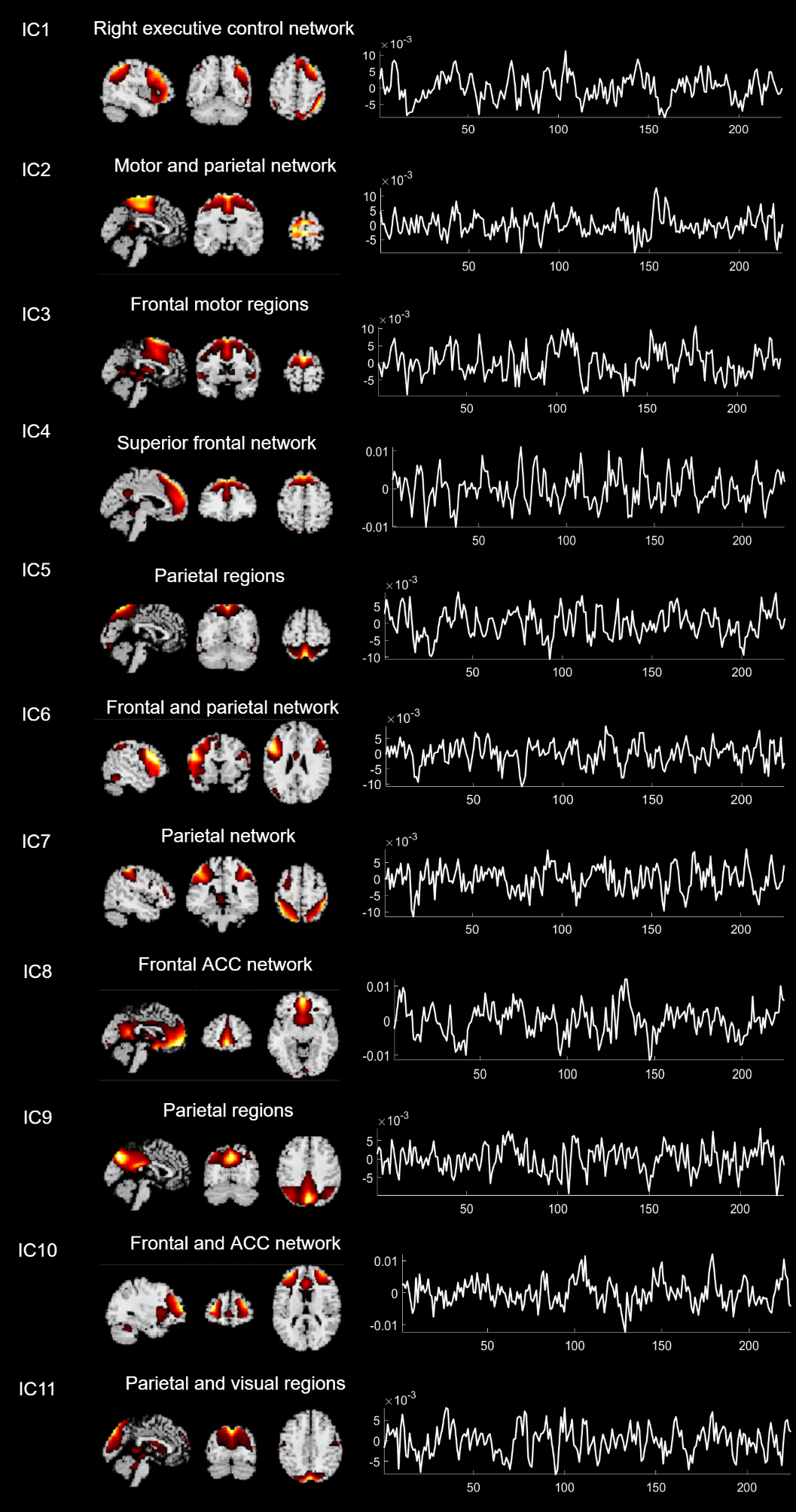


**Figure S7**. Spatial maps (*Z*-threshold > 1.0, in the left panel) and time series (in the right panel) for selected independent components of the mean for all participants.

Correlation analyses showed that SD and MSSD of BOLD signals were highly positively correlated for all ICs (see Table S1 for details), suggesting that SD- and MSSD-measured brain variability are highly consistent in rsfMRI data.

## Resting-state BOLD variability and individual differences in metacontrol

SD and MSSD of all ICs revealed negative correlations with RAT performance. SD of all selected components was not significantly related with RAT scores. We found a significant negative correlation between the MSSD of IC3 (i.e., frontal motor regions) and the RAT score (*r* = -0.350, *p*_uncorrected_ = 0.003, *p*_corrected_ < 0.05) (see Figure S8 and Table S2).

AUT flexibility and fluency scores were not significantly associated with brain variability.


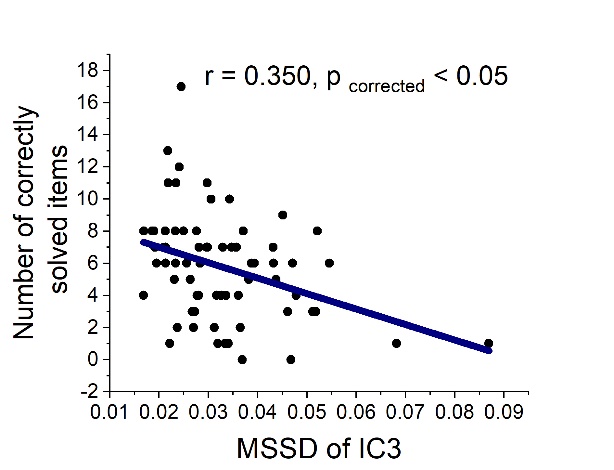


**Figure S8**. RAT performance was significantly negatively correlated with brain variability of the frontal motor regions (i.e., IC3). Brain variability was measured by MSSD.

**Table S1.** *Pearson correlations between brain variability measured by SD and MSSD.*

|  |  |  |  |  |
| --- | --- | --- | --- | --- |
| ICs |  | Correlation between SD and MSSD | |  |
|  |  | r | p |  |
|  |  |  |  |  |
| IC1 |  | **0.735** | **< .0001** |  |
| IC2 |  | **0.821** | **< .0001** |  |
| IC3 |  | **0.723** | **< .0001** |  |
| IC4 |  | **0.664** | **< .0001** |  |
| IC5 |  | **0.648** | **< .0001** |  |
| IC6 |  | **0.835** | **< .0001** |  |
| IC7 |  | **0.789** | **< .0001** |  |
| IC8 |  | **0.506** | **< .0001** |  |
| IC9 |  | **0.680** | **< .0001** |  |
| IC10 |  | **0.857** | **< .0001** |  |
| IC11 |  | **0.714** | **< .0001** |  |
|  |  |  |  |  |

*Note.* IC = Independent component, SD = standard deviation, MSSD = mean squared successive difference.

**Table S2.** *Correlations between brain variability measured by SD, MSSD, and metacontrol policies measured by RAT scores, AUT flexibility scores, and AUT fluency scores.*

|  |  |  |  |  |  |  |  |  |  |  |  |  |  |
| --- | --- | --- | --- | --- | --- | --- | --- | --- | --- | --- | --- | --- | --- |
| Brain variability measures | ICs |  | RAT scores | | |  | AUT flexibility scores | | |  | AUT fluency scores | | |
|  |  |  | r | p_uncorrected_ | p_corrected_ |  | r | p_uncorrected_ | p_corrected_ |  | r | p_uncorrected_ | p_corrected_ |
|  |  |  |  |  |  |  |  |  |  |  |  |  |  |
| SD | IC1 |  | -0.166 | 0.173 | 1.000 |  | 0.002 | 0.987 | 1.000 |  | -0.100 | 0.413 | 1.000 |
|  | IC2 |  | -0.163 | 0.181 | 1.000 |  | -0.205 | 0.091 | 1.000 |  | -0.215 | 0.076 | 1.000 |
|  | IC3 |  | -0.205 | 0.090 | 1.000 |  | -0.138 | 0.257 | 1.000 |  | -0.227 | 0.061 | 1.000 |
|  | IC4 |  | -0.162 | 0.184 | 1.000 |  | -0.088 | 0.471 | 1.000 |  | -0.121 | 0.323 | 1.000 |
|  | IC5 |  | -0.004 | 0.977 | 1.000 |  | -0.133 | 0.275 | 1.000 |  | -0.081 | 0.507 | 1.000 |
|  | IC6 |  | -0.202 | 0.096 | 1.000 |  | -0.146 | 0.231 | 1.000 |  | -0.061 | 0.620 | 1.000 |
|  | IC7 |  | -0.053 | 0.665 | 1.000 |  | -0.174 | 0.152 | 1.000 |  | -0.136 | 0.264 | 1.000 |
|  | IC8 |  | -0.002 | 0.988 | 1.000 |  | -0.040 | 0.744 | 1.000 |  | -0.119 | 0.331 | 1.000 |
|  | IC9 |  | -0.129 | 0.292 | 1.000 |  | -0.101 | 0.409 | 1.000 |  | -0.080 | 0.514 | 1.000 |
|  | IC10 |  | -0.274 | 0.023 | 1.000 |  | -0.244 | 0.044 | 1.000 |  | -0.262 | 0.030 | 1.000 |
|  | IC11 |  | -0.165 | 0.177 | 1.000 |  | -0.204 | 0.093 | 1.000 |  | -0.143 | 0.242 | 1.000 |
|  |  |  |  |  |  |  |  |  |  |  |  |  |  |
| MSSD | IC1 |  | -0.268 | 0.026 | 1.000 |  | -0.128 | 0.294 | 1.000 |  | -0.050 | 0.681 | 1.000 |
|  | IC2 |  | -0.301 | 0.012 | 1.000 |  | -0.260 | 0.031 | 1.000 |  | -0.174 | 0.153 | 1.000 |
|  | IC3 |  | **-0.350** | **0.003** | **0.033** |  | -0.251 | 0.037 | 1.000 |  | -0.150 | 0.218 | 1.000 |
|  | IC4 |  | -0.151 | 0.216 | 1.000 |  | -0.129 | 0.289 | 1.000 |  | -0.103 | 0.399 | 1.000 |
|  | IC5 |  | -0.181 | 0.136 | 1.000 |  | -0.196 | 0.106 | 1.000 |  | -0.017 | 0.892 | 1.000 |
|  | IC6 |  | -0.231 | 0.056 | 1.000 |  | -0.186 | 0.126 | 1.000 |  | -0.077 | 0.527 | 1.000 |
|  | IC7 |  | -0.149 | 0.223 | 1.000 |  | -0.267 | 0.027 | 1.000 |  | -0.253 | 0.036 | 1.000 |
|  | IC8 |  | -0.205 | 0.091 | 1.000 |  | -0.201 | 0.097 | 1.000 |  | -0.104 | 0.395 | 1.000 |
|  | IC9 |  | -0.188 | 0.122 | 1.000 |  | -0.242 | 0.045 | 1.000 |  | -0.099 | 0.417 | 1.000 |
|  | IC10 |  | -0.267 | 0.027 | 1.000 |  | -0.181 | 0.136 | 1.000 |  | -0.115 | 0.348 | 1.000 |
|  | IC11 |  | -0.193 | 0.113 | 1.000 |  | -0.222 | 0.067 | 1.000 |  | -0.050 | 0.683 | 1.000 |
|  |  |  |  |  |  |  |  |  |  |  |  |  |  |
|  |  |  |  |  |  |  |  |  |  |  |  |  |  |

*Note.* IC = Independent component, SD = standard deviation, MSSD = mean squared successive difference, RAT = Remote Associates Task, AUT = Alternate Uses Task, P corrected = Bonferroni corrected p value. Spearman correlation was used for correlation analyses.

# References

1. Speer, S. P. H., Smidts, A. & Boksem, M. A. S. Individual differences in (dis)honesty are represented in the brain’s functional connectivity: Robust out-of-sample prediction of cheating behavior. *bioRxiv* (2020) doi:10.1101/2020.05.12.091116.

2. Akbari Chermahini, S., Hickendorff, M. & Hommel, B. Development and validity of a Dutch version of the Remote Associates Task: An item-response theory approach. *Think. Ski. Creat.* **7**, 177–186 (2012).
